# Cell‐type specific impact of metformin on monocyte epigenetic age reversal in virally suppressed older people living with HIV

**DOI:** 10.1111/acel.13926

**Published:** 2023-09-07

**Authors:** Michael J. Corley, Alina P. S. Pang, Cecilia M. Shikuma, Lishomwa C. Ndhlovu

**Affiliations:** ^1^ Department of Medicine, Division of Infectious Diseases Weill Cornell Medicine New York City New York USA; ^2^ Hawaii Center for AIDS, John A. Burns School of Medicine University of Hawaii Honolulu Hawaii USA

**Keywords:** DNA methylation, epigenetic age, epigenetics, HIV, metformin, monocytes

## Abstract

The anti‐diabetic drug metformin may promote healthy aging. However, few clinical trials of metformin assessing biomarkers of aging have been completed. In this communication, we retrospectively examined the effect of metformin on epigenetic age using principal component (PC)‐based epigenetic clocks, mitotic clocks, and pace of aging in peripheral monocytes and CD8^+^ T cells from participants in two clinical trials of virologically‐suppressed people living with HIV (PLWH) with normal glucose receiving metformin. In a small 24‐week clinical trial that randomized participants to receive either adjunctive metformin or observation, we observed significantly decreased PCPhenoAge and PCGrimAge estimates of monocytes from only participants in the metformin arm by a mean decrease of 3.53 and 1.84 years from baseline to Week 24. In contrast, we observed no significant differences in all PC clocks for participants in the observation arm over 24 weeks. Notably, our analysis of epigenetic mitotic clocks revealed significant increases for monocytes in the metformin arm when comparing baseline to Week 24, suggesting an impact of metformin on myeloid cell kinetics. Analysis of a single‐arm clinical trial of adjunctive metformin in eight PLWH revealed no significant differences across all epigenetic clocks assessed in CD8^+^ T cells at 4‐ and 8‐week time points. Our results suggest cell‐type‐specific myeloid effects of metformin captured by PC‐based epigenetic clock biomarkers. Larger clinical studies of metformin are needed to validate these observations and this report highlights the need for further inclusion of PLWH in geroscience trials evaluating the effect of metformin on increasing healthspan and lifespan.

## Graphical abstract

People living with HIV exhibit immune dysfunction, chronic inflammation, and heightened signs of biological aging captured by epigenetic clocks. Here, we performed a post hoc cell‐type specific epigenetic age analysis of monocytes and CD8^+^ T cells using principal component‐based epigenetic clocks, epigenetic mitotic clocks, and a pace of aging epigenetic clock of two small metformin clinical trials in people living with HIV.

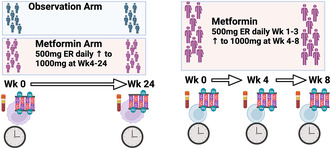

## 1 INTRODUCTION

Metformin is an FDA‐approved diabetes drug impacting aging biology (Barzilai et al., 2016), though metformin's effects on lifespan and healthspan remain controversial (Soukas et al., 2019). Moreover, few clinical studies have been conducted in potential target “accelerated” aging populations for metformin such as older, euglycemic, virally suppressed individuals living with HIV (PLWH), who exhibit heightened signs of biological aging (Cole et al., 2017; Guaraldi et al., 2009; Justice, 2010) due to HIV, chronic inflammation, antiretroviral therapy, and lifestyle effects (Deeks et al., 2013; Goulet et al., 2007; High et al., 2012). Epigenetic clocks detect HIV‐related aging effects (Gross et al., 2016; Horvath et al., 2018; Horvath & Levine, 2015; Leung et al., 2017; Shiau et al., 2021). However, no study has examined metformin's impact on these biomarkers in PLWH on adjunctive metformin. Here, we performed a post hoc cell‐type specific epigenetic age analysis using principal component (PC)‐based epigenetic clocks (Higgins‐Chen et al., 2022), epigenetic mitotic clocks (Teschendorff, 2020; Yang et al., 2016), and a pace of aging epigenetic clock (Belsky et al., 2022) of two small metformin clinical trials in euglycemic virally suppressed PLWH.

## 2 RESULTS AND DISCUSSION

**FIGURE 1:**
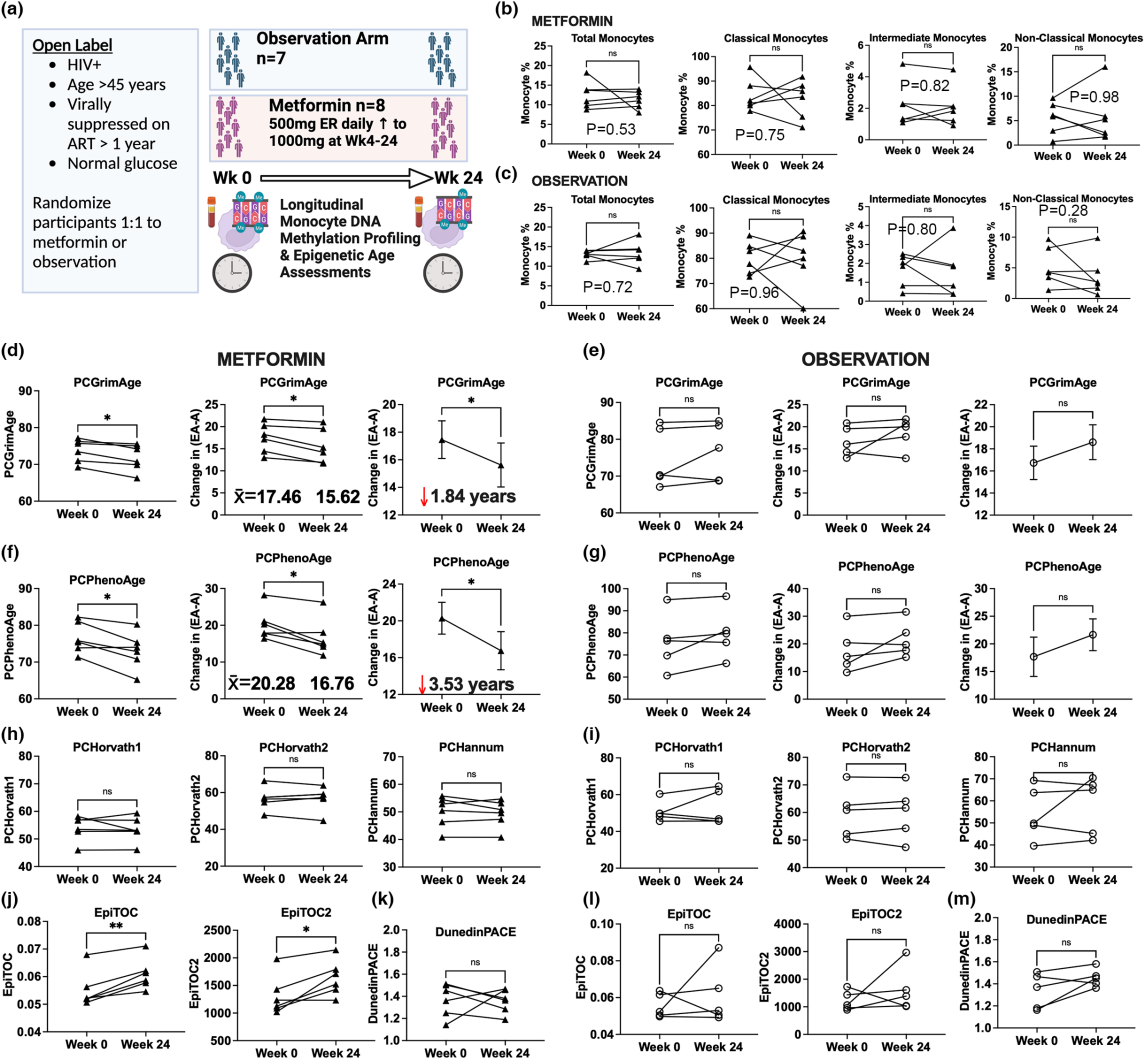
Epigenetic clock estimates in monocytes (a) Overview of 24 week clinical trial (b, c) Monocyte subsets did not significantly differ in participants in the metformin and observation arms. (d) PCGrimAge was significantly decreased in the metformin arm, *p* = 0.01(e) PCGrimAge did not significantly differ in the observation arm. (f) PCPhenoAge was significantly decreased in the metformin arm, *p* = 0.01. (g) PCPhenoAge did not significantly differ in the observation arm. (h, i) PCHorvath1, PCHorvath2, and PCHannum did not significantly differ in the metformin and observation arms. (j) EpiTOC and EpiTOC2 significantly increased in the metformin arm. (k) DunedinPACE did not significantly differ in the metformin arm. (l, m) EpiTOC, EpiTOC2, and DunedinPACE did not significantly differ in participants in the observation arm.

First, we examined epigenetic clocks of monocyte cells from a 24‐week clinical trial of 12 participants (time since HIV diagnosis ranged from 13 to 29 years) that were randomized 1:1 to receive either adjunctive metformin (500 mg extended release increasing to 1000 mg at Week 4 until 24 weeks) or observation. The study design of the retrospective analysis of a 24 week adjunctive metformin clinical trial is shown in Figure 1a and have been previously reported (Shikuma et al., 2020). The median chronological age did not significantly differ between groups for six participants randomized to the metformin arm (range, 51–60 years) and for five participants in the observational arm (range, 50–65 years). The study excluded participants with a history of diabetes. Hence, the baseline median fasting glucose of participants was 83 mg/mL (range, 71–104 mg/mL) in the metformin arm and 92 mg/mL (range, 75–105 mg/mL) in the observational arm.

We observed no significant differences in the percent of total monocytes, classical CD14^++^ CD16^−^ monocytes, intermediate CD14^++^ CD16^+^ monocytes, and nonclassical CD14^+^ CD16^++^ monocytes comparing Week 24 for both the metformin and observation groups using flow cytometry immunophenotyping (Figure 1b,c). To evaluate the impact of metformin on epigenetic clock biomarkers associated with age and mortality in total monocytes, we quantified DNA methylation (DNAm) levels at single nucleotide resolution using the MethylationEPIC platform from metformin and observation arm trial participants at Weeks 0 and 24. To minimize technical variation in epigenetic clock estimates that has been previously reported (Bose et al., 2014; Sugden et al., 2020), we harnessed a principal component (PC)‐based computational approach that increases reliability of epigenetic clocks tailored for use in longitudinal studies and clinical trials of interventions (Higgins‐Chen et al., 2022). We found that PCGrimAge, an epigenetic clock built on time‐to‐death and related to mortality and lifespan (Lu et al., 2019), was significantly decreased comparing Weeks 0–24 (*p* = 0.01; 95% Confidence Interval [CI], −3.03 to −0.64) by a mean of 1.84 years for participants in the metformin treatment group (Figure 1d). In contrast, PCGrimAge did not significantly differ for participants in the observation group (*p* = 0.29; 95% CI, −2.44 to 6.19) (Figure 1e). Additionally, we observed that PCPhenoAge, an epigenetic clock for lifespan and healthspan with a 1‐year increase associated with an increase in the risk of all‐cause mortality, was also significantly decreased comparing Weeks 0–24 (*p* = 0.01; 95% CI, −6.06 to −0.99) by a mean of 3.53 years for participants in the metformin treatment group (Figure 1f). PCPhenoAge did not significantly differ for participants in the observation group over 24 weeks (*p* = 0.13; 95% CI, −1.82 to 9.79) (Figure 1g). We did not observe any significant differences in chronological clocks including PCHorvath1 (Metformin: *p* = 0.65; 95% CI, −3.19 to 2.18; Observation: *p* = 0.47; 95% CI, −5.49 to 9.83), PCHorvath2 (Metformin: *p* = 0.90; 95% CI, −2.36 to 2.60; Observation: *p* = 0.82; 95% CI, −2.25 to 2.68), PCHannum (Metformin: *p* = 0.44; 95% CI, −2.89 to 1.46; Observation: *p* = 0.44; 95% CI, −8.37 to 15.8) over 24 weeks for participants in both the metformin treatment and observation arms (Figure 1h,i). Only GrimAge in the metformin treatment arm was significantly decreased comparing Weeks 0–24 (*p* = 0.02; 95% CI, −2.61 to −0.30) using original, non‐PC versions of epigenetic clocks (Figure S1). We did not observe any significant differences in the DunedinPACE pace of aging biomarker over 24 weeks for participants in both the metformin treatment and observation arms (Metformin: *p* = 0.88; 95% CI, −0.21 to 0.19 Observation: *p* = 0.11; 95% CI, −0.04 to 0.28) (Belsky et al., 2022). The lack of change due to metformin in first generation epigenetic clocks and DunedinPACE within monocytes may reflect cell‐type specific DNAm differences captured in the distinct construction of mortality epigenetic clocks versus chronological and pace of aging clocks.

Next, we utilized the DNAm dataset to examine whether metformin impacted monocytes DNAm mitotic clocks constructed from CpGs that map to gene promoters marked by the polycomb repressive complex 2 (PRC2) in human embryonic stem cells that estimate the relative stem cell division rate (Beerman et al., 2013; Teschendorff, 2020; Yang et al., 2016). We found that DNAm mitotic clock estimates EpiTOC and EpiTOC2 significantly increased comparing Weeks 0–24 for participants in the metformin group (EpiTOC: *p* = 0.003; 95% CI, −0.003 to 0.008; EpiTOC2: *p* = 0.01; 95% CI, 78.54 to 570.7) but did not differ for participants in the observation group (EpiTOC: *p* = 0.53; 95% CI, −0.017 to 0.027; EpiTOC2: *p* = 0.41; 95% CI, −797.6 to 1578) (Figure 1j,l).The implications of metformin's effect on the predicted increase in the mitotic clock in monocytes for aging improvement are not yet clear. Some studies suggest that aging leads to a decline in monocyte production and turnover, while others indicate an increase in monocyte turnover associated with age‐related immune dysfunction (De Maeyer & Chambers, 2021; He et al., 2018).

**FIGURE 2:**
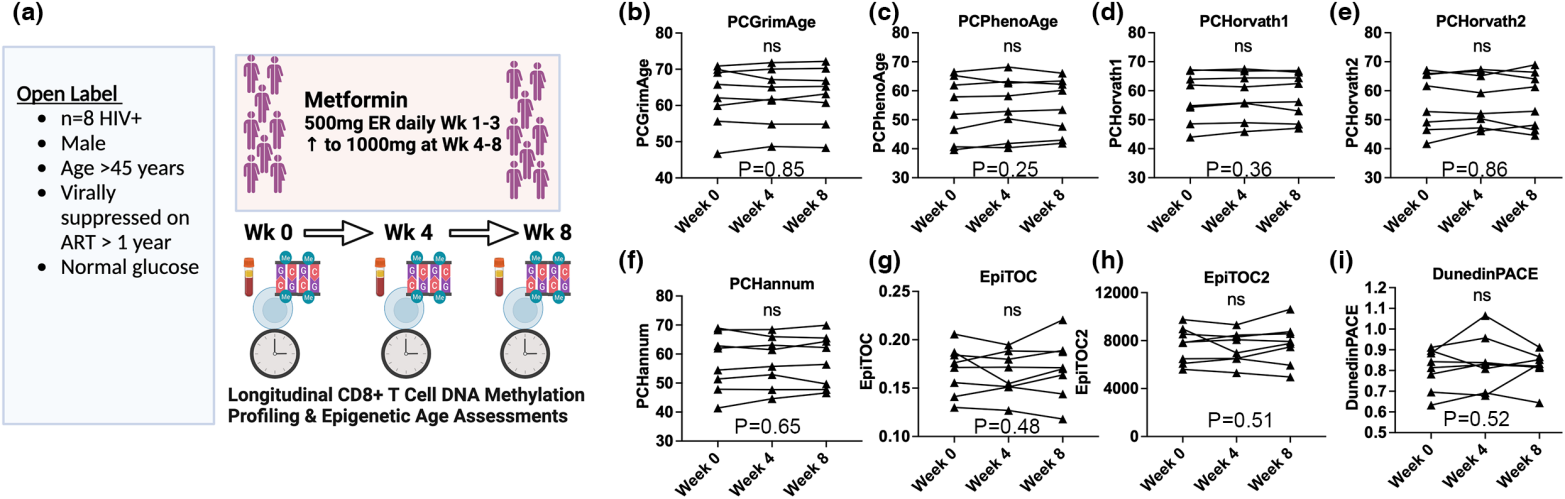
Epigenetic clock estimates in CD8^+^ T cells (a) Overview of 8 week clinical trial (b–I) No significant differences were observed in PCGrimAge, PCPhenoAge, PCHorvath1, PCHorvath2, PCHannum, EpiTOC, EpiTOC2, and pace of aging DunedinPACE in CD8^+^ T cells of participants comparing Weeks 0–4 and Week 8.

We next retrospectively examined epigenetic age of CD8^+^ T cells from a single arm 8‐week clinical trial that examined the effects of brief adjunctive metformin therapy in eight male euglycemic virally suppressed PLWH (Chew et al., 2021). We generated longitudinal DNAm from isolated CD8^+^ T cells for all participants at weeks 0, 4, and 8 (Figure 2a). Notably, we observed that PCGrimAge, PCPhenoAge, PCHorvath1, PCHorvath2, PCHannum, EpiTOC, EpiTOC2, and DunedinPACE did not significantly change in CD8^+^ T cells following either 4 weeks or 8 weeks of metformin therapy (Figure 2b‐i). Longer duration studies may be required to see an effect in T cells, which do not turnover as rapidly as monocyte cells. The effect of metformin on CD8^+^ T cells is not conclusive.

Observational epigenetic clock studies have reported conflicting metformin results on reducing epigenetic aging estimates (Li et al., 2022; Quach et al., 2017). A randomized metformin trial found no significant impact on epigenetic aging in overweight/obese breast cancer survivors (Nwanaji‐Enwerem et al., 2021), while another small trial combining metformin with human growth hormone and dehydroepiandrosterone in healthy men reported a reversal of epigenetic aging that persisted for 6 months posttreatment (Fahy et al., 2019). Our short report suggests metformin may benefit a subset of PLWH experiencing chronic systemic inflammation who may have the highest potential for epigenetic age reduction/reversal.

In summary, this brief report suggests adjunctive metformin in PLWH reduces mortality‐based epigenetic clocks in monocyte cells, potentially impacting accelerated and or accentuated aging—a major concern for PLWH due to increased age‐related comorbidities. It is crucial to further investigate metformin's cell‐type specific effects on monocytes through clinical trials. This report's limitations include a small sample size and the absence of follow‐up after metformin therapy to assess the durability of epigenetic clock changes in short‐lived monocyte cells. Our findings highlight the importance of pursuing more geroscience interventions that target biological aging in PLWH who stand to gain the most benefits and incorporating epigenetic clocks in clinical trials of metformin for aging to provide valuable insights into assessing treatment effects, understanding mechanisms, and longitudinal monitoring.

## AUTHOR CONTRIBUTIONS

Michael J. Corley and Lishomwa C. Ndhlovu conceived, designed and carried out experiments. Alina PS Pang contributed to data analysis and interpretation. Cecilia M Shikuma contributed biological specimens and clinical data. Michael J. Corley and Lishomwa C. Ndhlovu drafted the manuscript. All authors critically reviewed and edited the final version of the manuscript.

## FUNDING INFORMATION

L. C. N. and M.J.C. were supported during the draft of the manuscript in part by the National Institutes of Health (NIH) grant number UM1AI164559.

## CONFLICT OF INTEREST STATEMENT

L.C.N. reports grants from the NIH and has received consulting fees from work as a scientific advisor for AbbVie, ViiV Healthcare, and Cytodyn and for service on the Board of Directors of CytoDyn and has financial interests in Ledidi AS, all for work outside of the submitted work. All other authors declare no competing interests.

## Supporting information


Figure S1:

## Data Availability

The data from this study was submitted to the NCBI Gene Expression Omnibus (GEO) http://www.ncbi.nlm.nih.gov/geo/.
