# Supplementary material for: Cell‐type specific impact of metformin on monocyte epigenetic age reversal in virally suppressed older people living with HIV
Source: Aging Cell. 2023 Sep 7;23(1):e13926. doi: 10.1111/acel.13926 (PMC10776116; doi:10.1111/acel.13926)
Supplement: Supplementary file 1 — Figure S1: [file ACEL-23-e13926-s001.docx]

**Methods and Materials**

**Metformin 24 Week Clinical Trial Biospecimens**

De-identified cryopreserved peripheral blood mononuclear (PBMC) biospecimens were obtained from a previously conducted 24-week pilot clinical trial of adjunctive metformin involving 12 virally suppressed people living with HIV randomized 1:1 to metformin versus observation (OBS) (ClinicalTrials.gov: NC02383563). Detailed information on the study design, baseline subject characteristics, and primary outcomes have previously reported(*1*). The study randomized participants 1:1 to receive either metformin 500 mg extended release (ER) daily increasing to 1,000 mg ER daily at week 4 or to OBS. The study excluded individuals with history of diabetes, hepatitis B or C co-infection, uncontrolled medical condition or cancer, calculated creatinine clearance <60 mL/min, and recent history of illicit substance or alcohol use likely to interfere with the patient's ability to comply with protocol requirements. Baseline mean CD4 T cell count of participants did not significantly differ between groups and was 517.5 cells/mm^3^ (range, 277-793 cells/mm^3^) in the metformin arm and 759.4 cells/mm^3^ (range, 573-963 cells/mm^3^) in the observational arm. Baseline median CD4/CD8 ratio was less than 1 in both groups with the median CD4/CD8 ratio at 0.51 (range, 0.32-0.72) in the metformin arm and 0.85 (range, 0.43-1.38) in the observational arm. The time since HIV diagnosis for participants ranged from 13-29 years. The study was approved by the Committee on Human Subjects of the University of Hawaii and written informed consents were obtained from all participants. All participants also signed a separate informed consent document agreeing to the use of their biobanked specimens for future research.

**Metformin 8 Week Clinical Trial Biospecimens**

De-identified cryopreserved peripheral blood mononuclear (PBMC) biospecimens were obtained from a single-arm clinical trial of an 8-week course of metformin therapy in 8 euglycemic, virally suppressed people living with HIV. Detailed information on the study design, baseline subject characteristics, and primary outcomes have previously reported(*2*). Exclusion criteria included uncontrolled chronic medical conditions or cancers, acute illness within 2 weeks of entry, diagnosis of diabetes, history of chronic diarrhea, untreated hepatitis C coinfection, and laboratory parameters of hemoglobin <9.0 g/dL, absolute neutrophil count <1,000/mL, platelet count <50,000/mL, AST and ALT >5 × upper limit of normal (ULN), and creatinine clearance by Cockcroft and Gault of <60 mL/min. Adjunctive metformin extended-release dosing was 500 mg at entry to week 3, increasing to 1,000 mg at week 4 until end of study at week 8. The study was approved by the Committee on Human Subjects of the University of Hawaii and written informed consents were obtained from all participants. All participants also signed a separate informed consent document agreeing to the use of their biobanked specimens for future research.

**Peripheral total monocyte and CD8+ T cell enrichment and DNA isolations**

Cryopreserved peripheral blood mononuclear cells (PBMCs) were thawed in complete RPMI 1640 (cRPMI; HyClone) containing 10% fetal bovine serum (FBS) (Hyclone), 1% penicillin/streptomycin (HyClone), 10 mM HEPES (HyClone), 2 mM l-glutamine (Hyclone), and washed with 1 × phosphate-buffered saline (PBS; HyClone) before immunomagnetic enrichment. The EasySep Human Monocyte Isolation Kit (StemCell Technologies) was utilized according to the manufacturer’s protocol for immunomagnetic negative selection of total monocytes. The EasySep Human CD8+ T Cell Isolation Kit was utilized according to the manufacturer’s protocol for immunomagnetic negative selection of CD8+ T cells. The AllPrep DNA/RNA Mini Kit (Qiagen) was utilized to lyse cells in RLT Buffer and simultaneously purify genomic DNA and total RNA from enriched cell populations according to manufacturer’s instructions. DNA concentration was quantified and quality assessed utilizing a NanoDrop One Microvolume UV-Vis Spectrophotometer instrument (Thermo Fisher Scientific).

**Monocyte Imunophenotyping Flow Cytometry**

Cryopreserved peripheral blood mononuclear cells (PBMCs) were thawed in complete RPMI 1640 (cRPMI; HyClone) containing 10% fetal bovine serum (FBS) (Hyclone), 1% penicillin/streptomycin (HyClone), 10 mM HEPES (HyClone), 2 mM l-glutamine (Hyclone), and washed with 1 × phosphate-buffered saline (PBS; HyClone) before staining with Live/Dead aqua amine reactive dye (AARD; Invitrogen) or yellow amine reactive dye (YARD; Invitrogen). Cells were washed again with PBS supplemented with 2% FBS followed by panels of fluorochrome-conjugated anti-human mAbs to identify monocyte subsets: Beckman Coulter: ECD-conjugated anti-CD3 (Clone; UCHT1) mAb. BD Biosciences: PE-Cy7-conjugated anti-CD19 (SJ23C1), PE-Cy7-conjugated anti-CD20 (2H7), PE-Cy5-conjugated anti-CD38 (HIT2) mAbs. BioLegend: Brilliant Violet (BV) 605-conjugated anti-CD14 (M5E2), BV 711-conjugated anti-CD16 (3G8), APC-Cy7-conjugated anti-HLA-DR (L243), BV 510-conjugated anti-CD11b (ICRF44).

**Quantification of DNA methylation**

300 ng of DNA per sample were bisulfite converted using the EZ DNA Methylation kit (Zymo Research) according to the manufacturer’s instructions. Bisulfite-converted DNA samples were randomly assigned to a chip well on the Infinium HumanMethylationEPIC v1.0 BeadChip, amplified, hybridized onto the array, stained, washed, and imaged with the Illumina iScan SQ instrument to obtain raw image intensities. Raw Methylation EPIC array IDAT intensity data was loaded and preprocessed in the R statistical programming language (http://www.r-project.org) using the Sesame R package(*3*).

**Epigenetic Clock Calculations**

Epigenetic clock estimates, DNA methylation-based cell type deconvolution proportions, and epigenetic biomarkers were calculated using the online calculator (<https://dnamage.genetics.ucla.new>). We calculated epigenetic estimates for Horvath’s multi-tissue predictor DNAmAge based on 353 CpG sites (*4*), the Horvath skin-and-blood clock based on 391 CpG sites (*5*), Levine DNAmPhenoAge based on 513 CpG sites (*6*), Hannum’s clock based on 71 CpG sites (*7*), the Lu telomere length predictor, and DNA methylation based mortality risk assessment (GrimAge (*8*)) using the Horvath online calculator. Principal component-based epigenetic clock estimates were calculated utilizing an R script provided by Higgin-Chen et al. and 78,464 CpGs for each sample in a beta matrix(*9*). Mean imputation was utilized for missing values. Epigenetic mitotic clocks epiTOC(*10*) and epiTOC2(*10*) were calculated using the provided R-scripts available from zenodo. DunedinPACE pace of aging was calculated using the publicly available Github code(*11*).

**Statistics**

Paired T-tests were used to assess timepoint differences for metformin and observation groups in monocytes. Repeated measures ANOVA was utilized to analyze timepoint differences for CD8+ T cells.

**
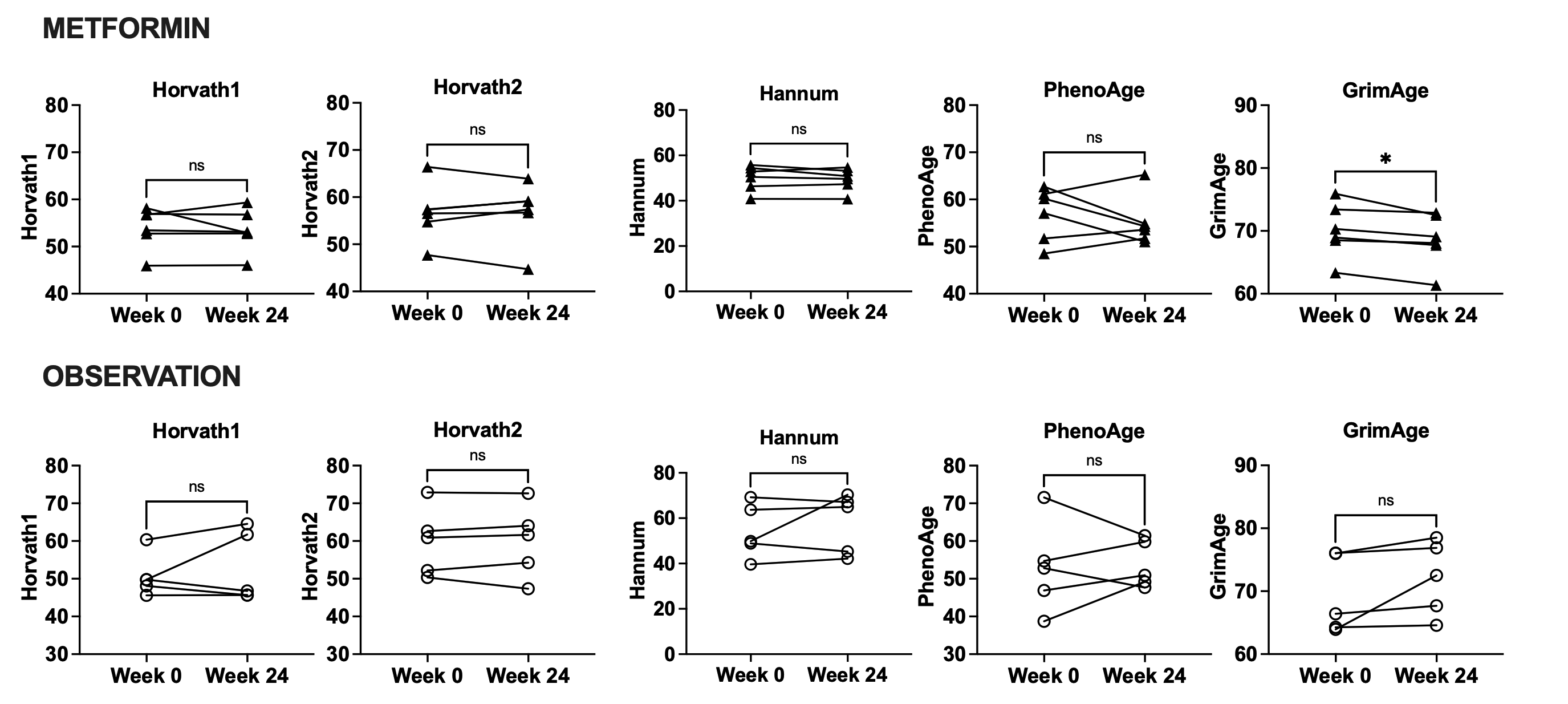
SUPPLEMENTAL FIGURE S1**

Non-PC version epigenetic clock estimates in monocytes from metformin and observation arm participants.

**REFERENCES**

1. C. M. Shikuma, G. M. Chew, L. Kohorn, S. A. Souza, D. Chow, I. N. SahBandar, E.-Y. Park, N. Hanks, L. M. A. Gangcuangco, M. Gerschenson, L. C. Ndhlovu, Short Communication: Metformin Reduces CD4 T Cell Exhaustion in HIV-Infected Adults on Suppressive Antiretroviral Therapy. *AIDS Res. Hum. Retroviruses*. **36**, 303–305 (2020).

2. G. M. Chew, A. J. P. Padua, D. C. Chow, S. A. Souza, D. M. Clements, M. J. Corley, A. P. S. Pang, M. M. Alejandria, M. Gerschenson, C. M. Shikuma, L. C. Ndhlovu, Effects of Brief Adjunctive Metformin Therapy in Virologically Suppressed HIV-Infected Adults on Polyfunctional HIV-Specific CD8 T Cell Responses to PD-L1 Blockade. *AIDS Res. Hum. Retroviruses*. **37**, 24–33 (2021).

3. W. Zhou, T. J. Triche Jr, P. W. Laird, H. Shen, SeSAMe: reducing artifactual detection of DNA methylation by Infinium BeadChips in genomic deletions. *Nucleic Acids Res.* **46**, e123 (2018).

4. S. Horvath, DNA methylation age of human tissues and cell types. *Genome Biol.* **14**, R115 (2013).

5. S. Horvath, J. Oshima, G. M. Martin, A. T. Lu, A. Quach, H. Cohen, S. Felton, M. Matsuyama, D. Lowe, S. Kabacik, J. G. Wilson, A. P. Reiner, A. Maierhofer, J. Flunkert, A. Aviv, L. Hou, A. A. Baccarelli, Y. Li, J. D. Stewart, E. A. Whitsel, L. Ferrucci, S. Matsuyama, K. Raj, Epigenetic clock for skin and blood cells applied to Hutchinson Gilford Progeria Syndrome and ex vivo studies. *Aging* . **10**, 1758–1775 (2018).

6. M. E. Levine, A. T. Lu, A. Quach, B. H. Chen, T. L. Assimes, S. Bandinelli, L. Hou, A. A. Baccarelli, J. D. Stewart, Y. Li, E. A. Whitsel, J. G. Wilson, A. P. Reiner, A. Aviv, K. Lohman, Y. Liu, L. Ferrucci, S. Horvath, An epigenetic biomarker of aging for lifespan and healthspan. *Aging* . **10**, 573–591 (2018).

7. G. Hannum, J. Guinney, L. Zhao, L. Zhang, G. Hughes, S. Sadda, B. Klotzle, M. Bibikova, J.-B. Fan, Y. Gao, R. Deconde, M. Chen, I. Rajapakse, S. Friend, T. Ideker, K. Zhang, Genome-wide methylation profiles reveal quantitative views of human aging rates. *Mol. Cell*. **49**, 359–367 (2013).

8. A. T. Lu, A. Quach, J. G. Wilson, A. P. Reiner, A. Aviv, K. Raj, L. Hou, A. A. Baccarelli, Y. Li, J. D. Stewart, E. A. Whitsel, T. L. Assimes, L. Ferrucci, S. Horvath, DNA methylation GrimAge strongly predicts lifespan and healthspan. *Aging* . **11**, 303–327 (2019).

9. A. T. Higgins-Chen, K. L. Thrush, Y. Wang, C. J. Minteer, P.-L. Kuo, M. Wang, P. Niimi, G. Sturm, J. Lin, A. Z. Moore, S. Bandinelli, C. H. Vinkers, E. Vermetten, B. P. F. Rutten, E. Geuze, C. Okhuijsen-Pfeifer, M. Z. van der Horst, S. Schreiter, S. Gutwinski, J. J. Luykx, M. Picard, L. Ferrucci, E. M. Crimmins, M. P. Boks, S. Hägg, T. T. Hu-Seliger, M. E. Levine, A computational solution for bolstering reliability of epigenetic clocks: Implications for clinical trials and longitudinal tracking. *Nat Aging*. **2**, 644–661 (2022).

10. A. E. Teschendorff, A comparison of epigenetic mitotic-like clocks for cancer risk prediction. *Genome Med.* **12**, 56 (2020).

11. D. W. Belsky, A. Caspi, D. L. Corcoran, K. Sugden, R. Poulton, L. Arseneault, A. Baccarelli, K. Chamarti, X. Gao, E. Hannon, H. L. Harrington, R. Houts, M. Kothari, D. Kwon, J. Mill, J. Schwartz, P. Vokonas, C. Wang, B. S. Williams, T. E. Moffitt, DunedinPACE, a DNA methylation biomarker of the pace of aging. *Elife*. **11** (2022), doi:10.7554/eLife.73420.
